# Structure and Property of Foam Glass-Ceramic Prepared by Copper Tailings

**DOI:** 10.3390/ma19081481

**Published:** 2026-04-08

**Authors:** Linyun Shi, Yingliang Tian, Mingfu Huang, Feng He, Yuanze Wang, Zhiyong Zhao

**Affiliations:** 1 School of Water Resources & Environmental Engineering, East China University of Technology, Nanchang 330001, China; 202560042@ecut.edu.cn; 2 Jiangxi Building Materials Scientific Research & Design Institute, Nanchang 330001, China; 3 School of Materials Science and Engineering, Beijing University of Technology, Beijing 100124, China; 4 State Key Laboratory of Materials Low-Carbon Recycling, Beijing 100124, China

**Keywords:** copper tailings, CaO/SiO_2_ ratio, foam glass-ceramics, thermal insulation

## Abstract

Large-scale reuse of copper tailings can mitigate environmental hazards and recover strategic elements; this work investigates the feasibility of producing foam glass-ceramics with high copper-tailing content (>70 wt%) by tuning the CaO/SiO_2_ ratio to couple melt viscosity and crystallisation. The comprehensive utilisation of these tailings helps mitigate environmental pollution and enhance resource efficiency. In this study, foam glass-ceramics with varying CaO/SiO_2_ ratios were synthesised through melt quenching followed by foaming heat treatment. The effects of different CaO/SiO_2_ ratios on the foaming behaviour, crystallisation, and microstructure were investigated using DSC, FTIR, viscosity, XRD, SEM, and CT. The results indicate that increasing the CaO/SiO_2_ ratio disrupts the three-dimensional network structure of the glass, which lowers the glass viscosity and influences the bubble size and distribution in the foam glass-ceramics. Additionally, the increased CaO content promotes crystal precipitation and enhances the compressive strength of the foam glass-ceramics. At a CaO/SiO_2_ mass ratio of 0.22, the foam glass-ceramics exhibited the lower bulk density (240 kg/m^3^) and thermal conductivity (0.07 W/m·K). The materials also demonstrated good water absorption and compressive strength. This study highlights the potential of using copper tailings in foam glass-ceramics to improve their overall performance, offering promising energy-saving and environmentally friendly solutions.

## 1. Introduction

The rapid development of industrial technology in countries worldwide has led to a year-on-year increase in the consumption of copper mineral resources [1]. Resulting in an increase in the global copper tailings of more than 129.7 billion tons, and low comprehensive utilisation of copper tailings is becoming increasingly serious [2]. The copper smelting industry has resulted in the accumulation of vast quantities of copper tailings (CTs), posing significant environmental and ecological concerns [3,4]. As of 2022, approximately 2.24 billion tons of copper tailings were estimated to be present in China, and the cumulative stockpile volume exceeded 15 billion tonnes [5]. These tailings typically contain substantial amounts of SiO_2_, Al_2_O_3_, Fe_2_O_3_ and CaO, together with minor metallic oxides. Owing to their silicate-rich composition, CTs exhibit a strong potential for construction material. However, the majority of copper tailings are still stockpiled or landfilled, leading to secondary pollution through heavy-metal leaching and long-term land occupation. The sustainable and high-value recycling of CTs has therefore become an urgent scientific and technological issue in the field of solid waste management [6,7].

In recent decades, extensive efforts have been made to recycle copper and other metallurgical tailings into building materials, such as concrete admixture [3,8], ceramics [9], and cementitious materials [10]. In the field of functional materials, copper tailings can be used to produce glass-ceramics, catalysts, abrasives, coatings, ceramic materials, and more [11]. It is hoped that copper tailings resources can be more fully utilised, larger-scale and high-value in the future, and will be widely promoted and applied in practical life [12]. While these approaches can partly alleviate environmental pressures, they generally involve low utilisation ratios and yield products with limited added value. The mechanisms of elements such as Fe, S, and Cu in copper tailings that contribute to the preparation of functional materials remain unclear. Foam glass and foam glass-ceramics, by contrast, offer a promising route to transform tailings into lightweight, thermally insulating and mechanically resilient materials [13]. Their porous structure allows the incorporation of large amounts of tailings—often exceeding 70 wt%—while simultaneously immobilising potentially hazardous elements within a stable silicate network.

Despite these advantages, several challenges hinder the preparation of high-quality foam glass-ceramics derived from copper tailings. The complex chemical composition of CTs often leads to an unpredictable crystallisation sequence and high melt viscosity, both of which severely restrict bubble formation and stability during foaming. Moreover, excessive crystallisation or inappropriate viscosity may cause the collapse or coalescence of pores, resulting in a heterogeneous microstructure and deteriorated mechanical performance. Understanding the coupling between glass composition, melt rheology, crystallisation behaviour and foaming dynamics is thus crucial for optimising both structure and properties.

Among various compositional factors, the CaO/SiO_2_ ratio plays a pivotal role in determining the polymerisation degree of the silicate network, thereby influencing viscosity and crystallisation kinetics. Increasing CaO content tends to depolymerise the network, reduce viscosity, and facilitate the formation of crystalline phases such as wollastonite or diopside. A rational adjustment of the CaO/SiO_2_ ratio is therefore expected to regulate the crystallisation–foaming balance and enhance the structural uniformity of foam glass-ceramics.

In this work, copper tailings were utilised as the principal raw material (above 70 wt%) to fabricate foam glass-ceramics. The CaO/SiO_2_ ratio was systematically varied to investigate its effects on the thermal behaviour, viscosity, crystallisation process, and pore evolution. The relationships among composition, microstructure, and properties were comprehensively analysed, aiming to provide both a fundamental understanding of the structure–property correlation and a practical strategy for the high-value recycling of copper tailings. It was shown that the iron (Fe) in copper tailings encourages the crystallisation of Fe-rich (Ca, Fe)SiO_3_ and that CaO/SiO_2_ has a significant role in controlling the pore structure of foam glass-ceramics.

## 2. Experimental Procedure

### 2.1. Design and Preparation

**Figure 1:**
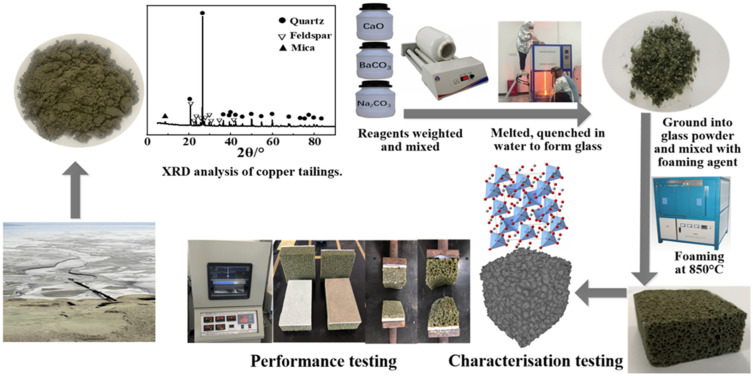
Foam glass-ceramic prepared schematic diagram.

As shown in Figure 1 and Table 1, the CTs, which are composed of quartz, feldspar and mica, and rich in all types of principal oxides (e.g., SiO_2_, Al_2_O_3_, and K_2_O), were used as raw materials. The oxide composition of the CT, determined by X-ray fluorescence, is listed in Table 1. The specimens with CaO/SiO_2_ mass ratios of 0.13, 0.18, 0.22 and 0.26 were prepared using 74–82 wt% copper tailings, named as FG1 to FG4 (Table 2). In addition, analytical-grade chemical powders such as calcium oxide, sodium carbonate, aluminium oxide, and barium carbonate were used to regulate the composition. GB 800 mesh silicon carbide (SiC) was added as a foaming agent. The raw materials and reagents were weighed and mixed in the proportions shown in Table 2 with a ceramic mixer. The mixed materials were melted and clarified at 1450 °C, then quenched in water to form glass slag. A foaming agent was added to the glass slag, which is then ground to a particle size of less than 75 μm, distributed, and sintered at a pre-set temperature, as Figure 1 shows.

The mixtures were melted at 1450 °C for 2 h and quenched using water, and then the as-prepared glass was uniformly ground with 0.5 wt% SiC. After sieving by 200 mesh, the powders were moved into ceramic crucibles and heated from room temperature to 850 °C at a rate of 10 °C/min, and the heat treatment duration was 30 min or 60 min. Specimens were cooled to ambient temperature along with the furnace.

### 2.2. Testing and Characterisation

The differential scanning calorimetry (DSC, NET NETZSCH-Gerätebau GmbH, Selb, Germany) curves of specimens were obtained using a synchronous thermal analyser. Measurement was conducted with a heating rate of 10 K/min and a gas flow rate of 20 mL/min in the air from 30 °C to 1000 °C, and the references were Al_2_O_3_ powder and Al_2_O_3_ pan. XRD tests were performed using an X-ray diffractometer (Bruker D8 ADVANCE, Bruker Corporation, Billerica, MA, USA) with Cu Kα radiation for 2θ ranging from 10° to 70° at a scanning rate of 8°/min. The microstructure of the glass-ceramic specimens was characterised using scanning electron microscopy (Regulus8100, Hitachi High-Technologies, Tokyo, Japan) after specimens were etched with a 5 vol% hydrofluoric acid solution. X-Ray Tomography tests were performed using Computed Tomography (GE Vtomex s, GE Sensing & Inspection Technologies, Billerica, MA, USA, and VG studio analysis software [M10.1]) at scanning voltage: 100 kV, current: 120 μA, and resolution: 10.5 μm. By combining high-temperature data with low-temperature data, the viscosity–temperature correlation curve is obtained. The high-temperature viscosity of glass melt was measured by the rotating spindle method. The Brookfield rheometer (DV2T; Brookfield Engineering Laboratories, Middleboro, MA, USA) was calibrated by standard silicone oil (9.5, 47.8, and 96.7 mPa·s) at a rotational speed of 100 rpm. The softening point temperature of glass (107.65 dPa·s) was determined by the Littleton method. The upper (1013 dPa·s) and lower (1014.5 dPa·s) limit annealing temperatures of glass were determined by GB/T 43873-2024 [14]. The test method for upper and lower annealing temperatures of ultra-thin glass. A glass fibre (0.6–0.8 mm diameter) is heated at 5 °C/min under a constant load. The upper limit is the temperature where elongation stops (speed < 0.01 mm/min), and the lower limit is where elongation reaches 0.4–0.6 mm/min. All viscosity data were fitted by the Vogel–Fulcher–Tammann (VFT) equation. A Cd-DR (J) 3030 thermal conductivity tester (Xiangtan Xiangyi Instruments and Meters Co., Ltd., Xiangtan, China) was used to test the thermal conductivity by the test method of Chinese standard GB/T 10294-2008 [15]. Samples of 400 mm × 400 mm × 20 mm from the same batch were subjected to three tests; statistical analysis produced a stable value, and each sample’s test result was the average of the two experiments. Thermal insulation was measured using the guarded hot plate method according to GB/T 10294-2008. This absolute method uses a guarded hot plate apparatus to establish steady-state one-dimensional heat flow through a plate-like specimen. The test specimen is placed between hot and cold plates. By measuring the electrical power input, temperature difference, and specimen thickness at thermal equilibrium, the thermal conductivity is calculated according to Fourier’s law. The bulk densities of the samples that were sintered were obtained by their mass-to-volume ratio. water absorption and compressive strength by the test method of Chinese standard JG/T 287-2013 [16] and GB/T 17671-2021 [17]. A full-scale mock-up undergoes 80 heat–rain cycles (heating to 70 °C, water spray), followed by 5 heat–cold cycles (50 °C to −20 °C). After conditioning, the system is inspected for cracks, bulging, or detachment, and the tensile bond strength is measured. GB/T 17671-2021 is the test method used to measure cement mortar strength (ISO method); the pressure exerted on the compressed area of the specimen when it reaches failure is calculated based on a uniform loading rate of 2400 N/S ± 200 N/S.

## 3. Results and Discussion

### 3.1. Performance Characteristics of CT Glass

**Figure 2:**
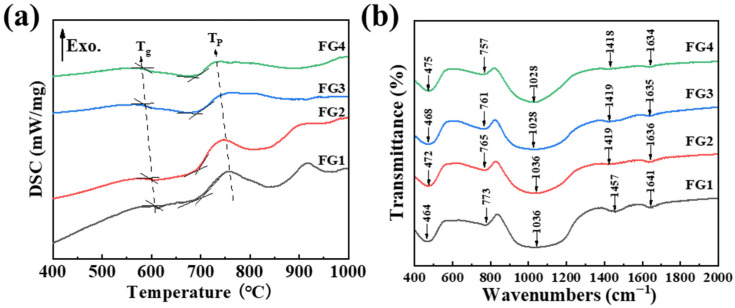
(**a**) DSC curves and (**b**) FT-IR of all as-prepared specimens.

Figure 2a shows the DSC curves of the parent glasses with different CaO/SiO_2_ ratios. All samples exhibit a distinct glass transition (Tg) followed by a single exothermic crystallisation peak (Tp). Both Tg and Tp decrease from 612 °C to 580 °C and from 760 °C to 732 °C, slightly with increasing CaO content, indicating that Ca^2+^ ions depolymerise the silicate network and reduce the viscosity of the melt.

To further investigate the influence of the CaO/SiO_2_ ratio on the glass network, Fourier-transform infrared (FTIR) spectroscopy was performed. Figure 2b shows several characteristic absorption bands at approximately 464–475 cm^−1^, 757–772 cm^−1^, and 1028–1036 cm^−1^. Other regions of the spectrum display a limited number of absorption bands. Table 3 shows the vibration type corresponding to each band. The bands near 470 cm^−1^ correspond to the bending vibration of the Si-O-Si bond in [SiO_4_]-tetrahedron [18,19], and 780 cm^−1^ are attributed to bending vibrations of Al-O-Al bonds in [AlO_4_] tetrahedrons [20]. The peak at 900–1200 cm^−1^ is associated with the asymmetrical stretching vibration of the Si–O–Si bond in [SiO_4_] tetrahedron, combined with a different number of bridging oxygens [21,22]. 1420–1460 cm^−1^ is the symmetric stretching vibration of the Si–O–Si bond generated by network-modified ions such as Ca/B [14], The band around 1640 cm^−1^ is related to molecular water or hydroxyl groups, such as Si-OH [23]. The FT–IR spectra of samples with different CaO/SiO_2_ compositions exhibit consistent characteristic absorption peaks. The internal absorption intensity of the wide band in the range of 900–1200 cm^−1^ and 470 cm^−1^ shows a relatively obvious change. The reason is that SiO_2_ accounts for the largest proportion in the glasses, the change in the content of CaO will cause the Si–O–Si bond in the range of 900–1200 cm^−1^ and 470 cm^−1^ to be more easily affected [24]. In addition, the band of 900–1200 cm^−1^ shifts to a lower wavenumber with the increase in CaO content (as shown in Figure 2b), which also indicates that the stability of the glass network structure becomes weakened. The reason can be explained by the well-known modified Hooke’s Equation [25].

**Figure 3:**
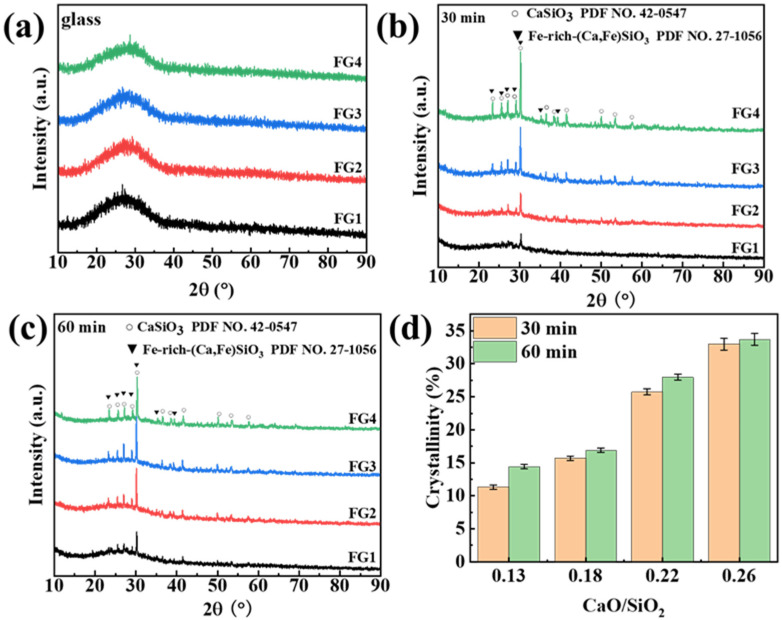
(**a**) XRD patterns of FG specimens, heat-treated at 850 °C for (**b**) 30 min, (**c**) 60 min, and (**d**) crystallinity of foam glass-ceramics calculated by Jade 6.0.

The foaming performance is primarily influenced by the viscosity and crystallisation ability of the glasses. Figure 3 presents the X–ray diffraction (XRD) patterns of both the as-prepared glasses and the heat-treated specimens. The slight narrowing of the halo with increasing CaO content suggests enhanced short-range ordering (Figure 3a), consistent with the FTIR results indicating network depolymerisation and local clustering of Ca–O units. No crystalline peaks were detected, confirming the successful vitrification of copper tailings. In contrast, for the heat-treated mixture of the as-prepared glass and foaming agent, distinct diffraction peaks appear, signifying the formation of crystalline phases such as wollastonite-1A (CaSiO_3_) and Fe-rich (Ca, Fe)SiO_3_. The formation of Fe-rich (Ca, Fe)SiO_3_ is attributed to the presence of Fe_2_O_3_ (approximately 2.51 wt%) in the copper tailings (Table 1). Additionally, the crystallinity of the samples increased with the CaO/SiO_2_ ratio, while the foaming duration showed negligible influence on crystallinity. As shown in Figure 3d, with increasing CaO/SiO_2_ ratio, the crystallinity increased from 11.33% to 32.97% for a foaming duration of 30 min, and from 14.40% to 33.70% for a foaming duration of 60 min, respectively. The wollastonite-1A phase in foamed glass-ceramics typically exhibits relatively higher volume stability [26,27]. This phenomenon can be attributed to the sulfides in copper tailings (CT), which induce nucleation of wollastonite, thereby enhancing the stability of the precipitated crystal phase and improving the crystallisation ability. This process extends the retention duration and modifies the pore structure. The formation of Fe-rich (Ca, Fe)SiO_3_ indicates that the iron (Fe) components in CT promote the crystallisation of foam glass, leading to the formation of stable crystal phases. CT contains pyroxene and other alkali metal silicate minerals. The [Si (Al)O_4_] tetrahedral chains in these silicate minerals are doped with cations, which can reduce the binding energy of valence bonds and, consequently, lower the liquid temperature. Additionally, Fe^3+^ in the copper tailings can replace Si^4+^ in the tetrahedral network, promoting crystallisation and lowering the crystallisation temperature (T_p_). Furthermore, sulfides, in copper tailings, act as nucleating agents for pseudo-wollastonite, allowing the pseudo-wollastonite crystal phase to precipitate at a lower temperature [28,29].

In the foaming process of foam glass-ceramic, controlling the liquid viscosity is crucial, as it significantly influences the thermal and mechanical properties, as well as the microstructure of the resulting samples. The viscosity (η) is directly related to the chosen foaming temperature, which determines the maximum stability of the foam and subsequently governs the shape, size, and uniformity of the pores. At relatively high foaming temperatures, η becomes very low (i.e., <105 dPa·s), making it difficult to maintain a stable foam structure. For the foam glass-ceramics, the decrease in viscosity can lead the gas to break through the barrier formed by the glass phase and escape, and the glass phase can also flow into some pore structures and fill them, ultimately causing the collapse of the sample [30,31]. Conversely, at low temperatures, η increases, which hinders the formation of well-foamed samples and requires sufficient pressure within the bubbles to overcome the high surface tension of the silicate glass melt.

**Figure 4:**
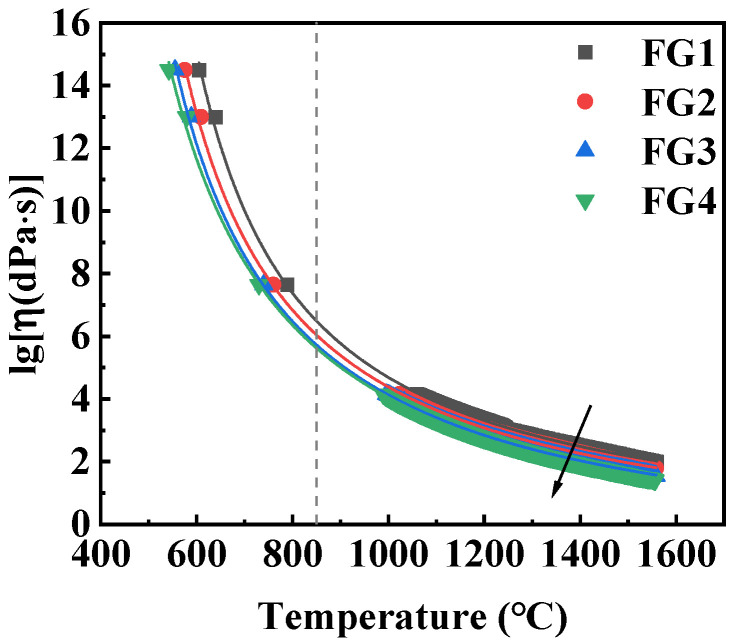
Temperature-dependent viscosity curves of as-prepared glasses.

Selecting an appropriate firing temperature is critical for foam glass production, as it is directly related to both the glass viscosity and the expansion resulting from the gas released by the decomposition of the foaming agent. In fact, within the typical working temperature range of the glass, the viscosity (ranging from 10^7.6^ to 10^4^ dPa·s) is sufficiently low to allow the gas generated to expand effectively [32]. The Vogel–Fulcher–Tammann (VFT) equation is used to express the dependence of the viscosity of glass on temperature [33]. Because the VFT equation has been proven to provide a good fitting tool, relevant data, and empirical curves for silicate glass, the VFT equation has the advantage of simplicity and wide applicability, so it has always been the most widely used equation to study the melting of silicate glasses.

(1) $$\begin{array}{c} lg\eta =A+\frac{B}{T-T_{0}} \end{array}$$

where η is the viscosity of the glass melt, T is the temperature, and A, B, and T_0_ are the parameters of the VFT equation. Among these, T_0_ is the temperature at which the configurational entropy disappears, A is the pre-exponential factor, and B is the rate at which viscosity increases as temperature drops. Figure 4 illustrates the viscosity-temperature dependence and the fitting curve for the glass. The corresponding VFT parameters are listed in Table 4. Increasing CaO/SiO_2_ markedly decreases the viscosity over the entire temperature range, as Ca^2+^ acts as a network modifier that breaks Si–O–Si bridges and enhances melt fluidity. At 850 °C, η decreases from 10^6.5^ to 10^5.6^ dPa·s with increasing CaO content, which is favourable for bubble growth during foaming while still maintaining sufficient viscous strength to prevent pore coalescence.

According to Stokes’ law, the rise and removal rate of bubbles in the melt is inversely proportional to the viscosity of the melt. Therefore, when the viscosity of the melt is reduced, it facilitates the rapid movement of gas, thereby promoting the completion of the foaming process. As the CaO/SiO_2_ ratio increases, the crystallinity of wollastonite also increases, while the glass viscosity gradually hinders the further growth of pores. During crystallisation, the high viscosity of the liquid phase requires sufficient pressure within the bubbles to overcome the strong surface tension of the silicate glass melt. This high surface tension makes it difficult for the pores to expand, thus limiting effective foaming.

### 3.2. Effect of the CaO/SiO_2_ Ratio on the Foaming Performance of Glass-Ceramic

**Figure 5:**
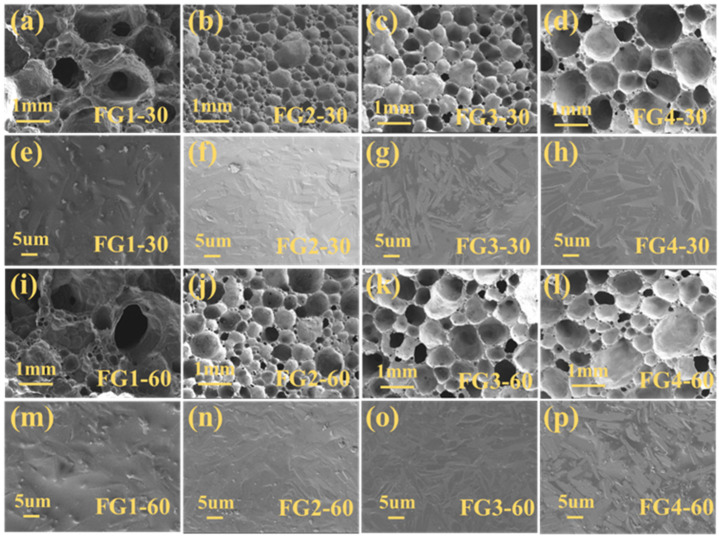
The SEM of foam glass-ceramics at 850 °C for (**a**–**h**) 30 min and (**i**–**p**) 60 min.

The pore structure picture of CT foam glass-ceramics with different CaO/SiO_2_ ratios is shown in Figure 5. The obtained foam glass-ceramics exhibit a closed-cell, three-dimensional porous structure without visible cracks. These holes are surrounded by columns having uneven cell wall thickness. For FG2, FG3 and FG4 specimens sintered for 30 min, foam glass-ceramics with an average pore size of 0.5 ± 0.2 mm, 0.8 ± 0.3 mm and 1 ± 0.5 mm. For FG2, FG3 and FG4 specimens sintered for 60 min, foam glass-ceramics with an average pore size of 0.8 ± 0.3 mm, 1 ± 0.5 mm and 1.5 ± 0.5 mm. Those specimens had good bubble growth stability with an average pore size of 1 mm when sintered for a prolonged sintering duration. Due to the extended holding time, the reaction between oxygen in the air and the silicon carbide particles immersed in the glassy liquid phase was accelerated, resulting in the generation of a substantial volume of gas. Owing to the surface tension of the glassy liquid phase, the generated bubbles grow into large bubbles [34].

For an FG2 specimen with a CaO/SiO_2_ ratio of 0.18, optimal homogeneity and the smallest average pore size (0.8 ± 0.3 mm) were obtained. FG1 specimens have a CaO/SiO_2_ ratio of 0.13, resulting in large holes and collapses in the sintered material. The macro-morphology of the foam glass-ceramics strongly depends on the CaO/SiO_2_ ratio. Samples with a lower CaO/SiO_2_ ratio (FG1–FG2) exhibit incomplete foaming and relatively dense microstructures, owing to their high melt viscosity, which restricts gas expansion. In contrast, excessive CaO (FG4) leads to bubble coalescence and collapse due to over-reduced viscosity. The sample FG3 shows the most uniform and stable foam structure, indicating an optimal viscosity window conducive to balanced bubble growth and structural rigidity.

SEM of CT foam glass-ceramics with different CaO/SiO_2_ ratios in Figure 5 shows that wollastonite crystals are uniformly distributed in the glass matrix in short columnar interspersions. The crystals of the FG4 specimen were thicker, increased in size, and grew with longer holding time. Since copper tailings mainly contain Si without Ca, the increase in CaO/SiO_2_ will reduce the amount of copper tailings. Copper tailings have the effect of lowering glass viscosity and promoting crystallisation of low CaO/SiO_2_, at a 0.22 CaO/SiO_2_ ratio, stable foaming with the lowest bulk density of CT foam glass-ceramic at 76.6% utilisation was achieved.

**Figure 6:**
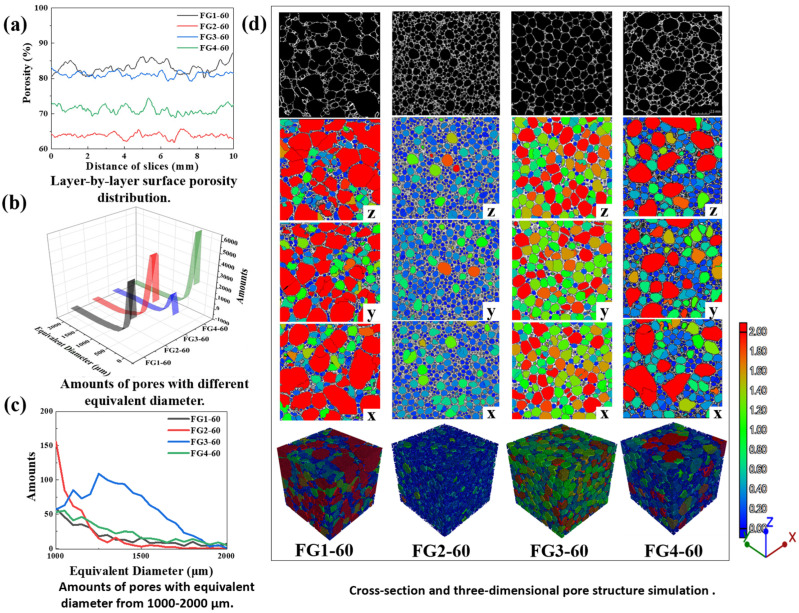
The CT of foam glass-ceramics at 850 °C for 60 min, (**a**) layer-by-layer surface porosity distribution, (**b**) volume of pores with different equivalent diameters of pores, and (**c**) volume of pores with regional equivalent diameters of pores, and (**d**) cross-section and three-dimensional pore structure simulation.

Three-dimensional X-ray computed tomography (CT) was employed to quantitatively characterise the internal pore architecture of the foamed glass-ceramics after sintering at 850 °C for 60 min. The reconstructed images (Figure 6) and corresponding pore size distribution curves were obtained using VG Studio Max software (Version 3.0, Volume Graphics GmbH, Heidelberg, Germany). The analyses focused on several key parameters, including porosity, average pore diameter, pore size distribution, pore sphericity, and the layer-by-layer variation in pore volume fraction.

The porosity and pore size distribution of the foam glass-ceramics are presented in Figure 6a–c. As shown in Figure 6a, FG1-60 and FG3-60 exhibit high porosities exceeding 80%, whereas FG4-60 and FG2-60 show lower porosities of approximately 70% and 65%, respectively. Figure 6b,c shows the pore size distributions obtained from CT quantitative analysis. Due to the resolution and segmentation limitations of the CT data, only pores with equivalent diameters <2000 μm were included in the statistical analysis. Larger pores (>2000 μm) were identified qualitatively from the three-dimensional reconstructed images, where they are highlighted in red and referred to as through-pores. Within the measurable size range, FG2-60 is dominated by small pores (<500 μm), resulting in a dense structure and low porosity. FG3-60 exhibits a relatively uniform pore size distribution, with a high fraction of medium-sized pores (1000–2000 μm) and a limited number of small pores acting as fillers. In contrast, FG1-60 and FG4-60 show heterogeneous pore size distributions, accompanied by abundant large through-pores (>2000 μm), indicating unstable pore growth and coalescence during foaming.

As revealed by the CT reconstructions (Figure 6), the pore morphology and spatial distribution are strongly influenced by the CaO/SiO_2_ ratio. FG3-60 exhibits the most uniform and stable cellular structure, characterised by high pore sphericity and thin, continuous pore walls. This morphology indicates an optimal balance between melt viscosity reduction and crystallisation during foaming. In contrast, FG1-60 and FG4-60 display irregular pore morphologies and pronounced structural heterogeneity, which can be attributed to either insufficient or excessive depolymerisation of the glass network, leading to unstable gas retention and pore coalescence.

The layer-by-layer porosity mapping (Figure 6a) further reveals the evolution of pore uniformity across the sample thickness. FG3 maintains a nearly constant porosity throughout the depth, implying balanced gas release and melt viscosity. Conversely, FG1 and FG4 show significant porosity gradients, suggesting uncontrolled gas escape near the surface and local pore collapse. The cumulative pore volume curves (Figure 6b,c) demonstrate that the moderate CaO/SiO_2_ ratio (0.22) provides optimal coupling between gas evolution, viscosity, and crystallisation rate. Excess CaO accelerates crystallisation, which prematurely solidifies the matrix and suppresses further bubble growth, while insufficient CaO leads to overly viscous melts that hinder gas expansion. Moreover, the CT results combined with XRD analysis confirm that moderate crystallisation around the pore walls enhances mechanical integrity by providing a rigid skeleton that supports the cellular structure. Excessive crystallisation, as in FG4, promotes uneven solidification and weakens structural continuity. The well-organised honeycomb structure observed in FG3 corresponds to its lower bulk density (240 kg·m^−3^), lowest thermal conductivity (0.07 W·m^−1^·K^−1^), and highest compressive strength. These findings demonstrate that the controlled coupling of crystallisation, viscosity, and pore stability—achieved by optimising the CaO/SiO_2_ ratio—is essential for obtaining high-performance foam glass-ceramics derived from copper tailings.

These structural features can be rationalised by the foaming mechanism associated with SiC oxidation and the composition-dependent viscosity and crystallisation balance. The foaming process is governed by the oxidation of SiC, producing CO and CO_2_ gases according to SiC(s) + nO_2_(g)→SiO_2_(s) + CO(s) + nO_2_(g)→SiO_2_(s) + CO_2_/CO(g). The release of gases and their retention within the viscous melt depend critically on the melt viscosity and crystallisation rate. Increasing CaO depolymerises the network and lowers viscosity, which facilitates bubble expansion and bubble growth. However, excessive depolymerisation also promotes early crystallisation of Ca–Si–O phases, consuming the liquid matrix and impeding further expansion. Therefore, an appropriate CaO/SiO_2_ ratio yields a balanced interplay between gas evolution, viscosity, and crystallisation, leading to stable foaming.

At low CaO/SiO_2_ ratios, a highly polymerised silicate network results in elevated melt viscosity, which restricts gas-driven expansion during SiC oxidation and leads to incomplete foaming and heterogeneous pore structures. With increasing CaO content, Ca^2+^ ions act as network modifiers, depolymerising the Si–O–Si framework and reducing melt viscosity, thereby promoting bubble expansion and pore development.

Meanwhile, an elevated CaO/SiO_2_ ratio favours the precipitation of Ca–Si–O crystalline phases, mainly wollastonite and Fe-containing silicates. The formation of these crystalline phases, particularly in the later stage of foaming, contributes to the gradual stiffening of the glassy matrix. Moderate crystallisation enhances the mechanical stability of pore walls and suppresses excessive pore coalescence, whereas excessive CaO addition accelerates crystallisation, reduces the effective liquid phase, and limits further bubble growth, resulting in pore coarsening and structural instability.

Consequently, an optimal CaO/SiO_2_ ratio establishes a balanced viscosity–crystallisation window in which melt flow, gas release, and structural stiffening are reasonably synchronised. This balance enables stable pore evolution and a more uniform cellular architecture, consistent with the superior structural integrity observed for foam glass-ceramics with a CaO/SiO_2_ ratio of 0.22.

### 3.3. Performance of CT Foam Glass-Ceramic

**Figure 7:**
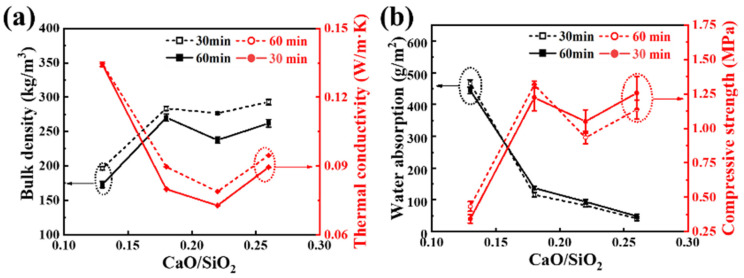
The performance of CT foam glass-ceramic; (**a**) bulk density and thermal conductivity; (**b**) water absorption and compressive strength.

Thermal conductivity is a key parameter in determining the thermal insulation performance of materials. The thermal conductivity of foam glass is influenced by solid conduction, gas conduction, thermal radiation, and convection. Figure 7a shows the variation in bulk density and thermal conductivity of the samples as the CaO/SiO_2_ ratio increases. As the CaO/SiO_2_ ratio increases, the bulk density first decreases and then increases. At a CaO/SiO_2_ ratio of 0.22, the minimum bulk densities are 280 kg/m^3^ (30 min) and 240 kg/m^3^ (60 min). The thermal conductivity follows a similar trend to that of the bulk density, with the minimum values being 0.0788 W/(m·K) (30 min) and 0.0728 W/(m·K) (60 min) at a CaO/SiO_2_ ratio of 0.22. These changes in bulk density and thermal conductivity are primarily influenced by the microstructure of the foamed glass, particularly the size and distribution of the bubbles. Excessively large bubbles can lead to convection under temperature gradients, which increases thermal conductivity. This explains why the thermal conductivity is higher when the CaO/SiO_2_ ratio is 0.26 compared to 0.22.

Water absorption is another important property of thermal insulation materials, as it impacts both performance and durability. Water has a thermal conductivity of 0.6 W/m·K, approximately 20 times higher than that of air (0.0026 W/m·K). If foam glass absorbs too much water, the voids become filled with water molecules, which reduces its overall thermal insulation performance. Additionally, when thermal insulation materials are exposed to cold temperatures, the water in the pores can freeze, potentially causing cracks and reducing the material’s service life. This is especially critical in colder regions. Therefore, it is crucial to keep the water absorption of thermal insulation materials at a low level. In this study, as the CaO/SiO_2_ ratio increases from 0.13 to 0.26, the water absorption decreases from 460 g/m^2^ to 430 g/m^2^ (30 min) and from 448 g/m^2^ to 50 g/m^2^ (60 min). This demonstrates that increasing the CaO content reduces the water absorption of foamed glass-ceramics. Samples with higher CaO/SiO_2_ show slightly increased absorption due to partial interconnection of pores, consistent with the CT reconstructions. These findings confirm that optimising pore morphology is crucial for achieving simultaneous thermal insulation and moisture resistance.

As a building thermal insulation material, the compressive strength of foam glass is critical because it affects the material’s service life, construction performance, and thermal insulation efficiency, ensuring the safety, energy efficiency, and comfort of the building. Figure 6b shows the trend of compressive strength for copper tailings foam glass as the CaO/SiO_2_ ratio increases. The compressive strength is influenced by factors such as foam structure, porosity, and microcrystalline structure, resulting in a complex trend. Considering the combined factors of bulk density, thermal conductivity, water absorption, and compressive strength, the sample with the best overall performance is obtained at a CaO/SiO_2_ ratio of 0.22.

As shown in Table 5, through the combination of copper tailings and CaO, the sintering temperature of foam ceramics is significantly reduced. Therefore, the cost of this experiment is lower and more environmentally friendly. As shown in the table, low density and porosity result in low strength values, but provide superior thermal insulation properties.

## 4. Conclusions

Foam glass-ceramics were successfully fabricated using more than 70 wt% copper tailings as the principal raw material, demonstrating the feasibility of transforming metallurgical waste into high-value functional materials. The influence of the CaO/SiO_2_ ratio on the thermal behaviour, viscosity, crystallisation, and foaming performance was systematically investigated. As the CaO/SiO_2_ ratio increases, both the crystallinity and thermal insulation performance of the foam glass-ceramics significantly improve. The foam glass-ceramics with a CaO/SiO_2_ ratio of 0.22 (60 min) exhibited the best overall performance, with optimal pore structure to achieve foaming stability, the lowest bulk density (240 kg·m^3^), and thermal conductivity (0.0728 W·m^1^·K^1^), as well as low water absorption (94 g·m^2^) and high compressive strength (1.04 MPa). By adjusting the CaO/SiO_2_ ratio, the microstructure of the foam glass can be effectively controlled to further optimise its thermal insulation properties. In summary, the CaO/SiO_2_ ratio is a key compositional parameter that governs the thermal, structural, and foaming behaviour of copper tailing-based glass-ceramics. The findings not only provide fundamental insights into the structure–property relationships of such systems but also demonstrate a sustainable pathway for the large-scale reutilisation of copper tailings in advanced glass-ceramic materials.

## Figures and Tables

**Table 1:** Composition of pre-desulfurized copper tailings (wt%).

Compounds	SiO_2_	CaO	Al_2_O_3_	MgO	K_2_O	Na_2_O	Fe_2_O_3_	TiO_2_	SO_3_	CuO	ZnO
CTs	72.67	3.24	11.52	0.56	7.05	0.28	2.51	0.27	1.45	0.09	0.07

**Table 2:** Composition of copper tailings foam glass-ceramic (wt%).

Sample	CaO/SiO_2_	CTs	SiO_2_	CaO	BaO	K_2_O + Na_2_O	Al_2_O_3_	SiC
FG1	0.13	81.32	59.1	7.68	4	15.81	17.37	0.5
FG2	0.18	78.58	57.1	10.28	4	15.81	17.05	
FG3	0.22	76.58	55.65	12.24	4	15.81	16.82	
FG4	0.26	74.65	54.25	14.11	4	15.81	16.60	

**Table 3:** FTIR absorption peaks and bands assignments of the glasses.

Wavenumber (cm^−1^)	Vibration Types	References
~470	Bending vibration of the Si-O-Si bond in [SiO_4_]-tetrahedron	[10,11]
~770	Bending vibrations of Al-O-Al bonds in [AlO_4_] tetrahedrons	[12]
900–1200	The asymmetrical stretching vibration of the Si-O-Si bond in [SiO_4_] tetrahedron, combined with a different number of bridging oxygens	[13,14]
~1420	Bending vibration of the Si-O-Si bond induced by Ca/B and other network-modifying ions	[14]
~1640	Bending vibration of O-H bonds	[15]

**Table 4:** Parameter of the Vogel–Fulcher–Tammann (VFT) equation.

	Parameter	A		B		T_0_	
No.		Value	Error	Value	Error	Value	Error
FG1		−0.90	0.005	3434.21	6.909	385.20	0.572
FG2		−1.22	0.007	3680.28	8.855	343.24	0.747
FG3		−1.55	0.002	3887.62	3.321	315.93	0.280
FG4		−1.90	0.003	4233.46	4.233	287.64	0.347

**Table 5:** Comparison of properties of foam ceramics.

Main Systems	Sintering Temperature (°C)	Density (g/cm^3^)	Porosity (%)	Water Absorption (%)	Compressive Strength (MPa)	References
Copper tailings: CaO/SiO_2_	850	0.24	82.10	0.39	1.04	Our manuscript
RWCP: WGP	890	0.83	66.68	8.27	2.39	[23]
Vitrification slag: biochar	1050	0.37	81.46	0.34	0.93	[35]
Coal gangue, diatomite, and dolomite	1230	0.41	74.86	1.48	1.64	[36]
Rud mud and granite scrap	1130	0.48	77.27	0.49	1.62	[37]
Asbestos tailings: coal fly ash	1180	1.52–1.70	30–40	0.1–0.4	8.81–19.2	[38]
Phosphorus tailings: coal gangue	1150	0.99	61.79	3.09	10.08	[39]
Graphite tailings	1200	1.36	—	0.35	12.03	[40]

## Data Availability

The original contributions presented in this study are included in the article. Further inquiries can be directed to the corresponding authors.

## References

[1] Wang Z., Li Z., Zou A., Pan Z. (2025). Green rebirth of copper tailings: From environmental burden to efficient utilization strategies. Environ. Prog. Sustain. Energy.

[2] Adrianto L.R., Pfister S., Hellweg S. (2022). Regionalized Life Cycle Inventories of Global Sulfidic Copper Tailings. Environ. Sci. Technol..

[3] Gupta R.C., Mehra P., Thomas B.S. (2017). Utilization of Copper Tailing in Developing Sustainable and Durable Concrete. J. Mater. Civ. Eng..

[4] Ahmari S., Zhang L. (2012). Production of eco-friendly bricks from copper mine tailings through geopolymerization. Constr. Build. Mater..

[5] (2012–2021). Chinese Bulk Industrial Solid Waste Comprehensive Utilization Industry Development Report.

[6] Jian S., Gao W., Lv Y., Tan H., Li X., Li B., Huang W. (2020). Potential utilization of copper tailings in the preparation of low heat cement clinker. Constr. Build. Mater..

[7] Tian X., Zhang H., Zhang T., Fernández C.A. (2020). Alkali-activated copper tailings-based pastes: Compressive strength and micro-structural characterization. J. Mater. Res. Technol..

[8] Chen S.C., Gao M.Y., Lin W.T., Huang C.Y., Chen W.C. (2024). Strength and microstructural development in concrete pavements by blended copper tailings powder and copper tailings sand. Mater. Today Commun..

[9] Oetomo D.S., Ramdhani R.F., Hermawan A. (2022). Feasibility Study for the Erection of a Ceramic Plant Made from Gold and Copper Mining Tailings in Timika District, Papua Province. Int. J. Res. Sci. Innov..

[10] Liu Y., Yi Y., Li C., Ma J., Yang Y., Zubaidaimu R. (2024). Study on the Mechanism of Gelling Performance of Sodium Carbonate-Activated Copper Tailings Based on Response Surface Method. Nonferrous Met. Eng..

[11] Zhong C., Gong J., Tan L. (2019). Modeling intraphase and interphase mass transfer limitations for NH_3_–SCR over Cu–ZSM–5. Chem. Eng. Sci..

[12] Wei L., Xu H., Wu J. (2025). A review of research progress on the resource utilization of copper tailing. J. Environ. Chem. Eng..

[13] Zhang L., Liang L., Li Y., Chen J., Cui Z., Qiao J., Zhang Z., Wang Z., Xu Q., Zhao C. (2024). Preparation of lightweight foam glass-ceramics from copper slag tailings: Secondary aluminum slag as pore-forming agent. Ceram. Int..

[14] (2024). Test Method for Upper and Lower Annealing Temperatures of Ultra-Thin Glass.

[15] (2008). Thermal Insulation-Determination of Steady-State Thermal Resistance and Related Properties-Guarded Hot Plate Apparatus.

[16] (2013). The Materials of External Thermal Insulation Systems Based on Insulated Decorative Panel.

[17] (2021). Test Method of Cement Mortar Strength (ISO Method).

[18] Yu L., Xiao H., Cheng Y. (2008). Influence of magnesia on the structure and properties of MgO-Al_2_O_3_-SiO_2_-F_−_ glass-ceramics. Ceram. Int..

[19] Gui H., Li C., Lin C., Zhang Q., Luo Z., Han L., Liu J., Liu T., Lu A. (2019). Glass forming, crystallization, and physical properties of MgO-Al_2_O_3_-SiO_2_-B_2_O_3_ glass-ceramics modified by ZnO replacing MgO. J. Eur. Ceram. Soc..

[20] Liu J., Luo Z., Lin C., Han L., Gui H., Song J., Liu T., Lu A. (2019). Influence of Y_2_O_3_ substitution for B_2_O_3_ on the structure and properties of alkali-free B_2_O_3_-Al_2_O_3_-SiO_2_ glasses containing alkaline-earth metal oxides. Phys. B Condens. Matter.

[21] Kalinkin A.M., Kalinkina E.V., Politov A.A., Makarov V.N., Boldyrev V.V. (2004). Mechanochemical interaction of Ca silicate and aluminosilicate minerals with carbon dioxide. J. Mater. Sci..

[22] ElBatal F.H., Marzouk M.A., ElBatal H.A. (2016). Optical and crystallization studies of titanium dioxide doped sodium and potassium silicate glasses. J. Mol. Struct..

[23] Qian B., Liang X., Yang S., He S., Gao L. (2012). Effects of lanthanum addition on the structure and properties of iron phosphate glasses. J. Mol. Struct..

[24] Liu J., Zou Q., Zhang Z., Zeng Q., Peng H., Wang Q., Chang Q. (2022). Research on mixed alkaline-earth effect in non-alkali glass substrates for TFT-LCDs. J. Non-Cryst. Solids.

[25] Han L., Liu J., Lin C., Gui H., Song J., Zhang Q., Li C., Luo Z., Liu T., Lu A. (2019). Sr^2+^/Y^3+^ co-doped MgO-Al_2_O_3_-SiO_2_-based glasses and transparent glass-ceramics: Crystallization, structure and properties. Ceram. Int..

[26] Yang H., Prewitt C.T. (1999). On the crystal structure of pseudowollastonite (CaSiO_3_). Am. Mineral..

[27] Ohashi Y. (1984). Polysynthetically-twinned structures of enstatite and wollastonite. Phys. Chem. Miner..

[28] Coruh S., Ergun O.N., Cheng T.W. (2006). Treatment of copper industry waste and production of sintered glass-ceramic. Waste Manag. Res..

[29] Barbieri L., Karamanov A., Corradi A., Lancellotti I., Pelino M., Rincon J.M. (2008). Structure, chemical durability and crystalliza-tion behavior of incinerator-based glassy systems. J. Non-Cryst. Solids.

[30] Ruan M., Tian Q., Zhang M., Wang C., Xu G., Cai J., Fu Q. (2024). Fabrication and characterization of foam ceramics from recycled waste concrete powder and waste glass powder. Constr. Build. Mater..

[31] Awoyera P.O., Britto B.F. (2020). Foamed concrete incorporating mineral admixtures and pulverized ceramics: Effect of phase change and mineralogy on strength characteristics. Constr. Build. Mater..

[32] Souza M.T., Maia B.G.O., Teixeira L.B., de Oliveira K.G., Teixeira A.H.B., de Oliveira A.P.N. (2017). Glass foams produced from glass bottles and eggshell wastes. Process Saf. Environ. Prot..

[33] Garca-Coln L.S., del Castillo L.F., Goldstein P. (1989). Theoretical basis for the Vogel-Fulcher-Tammann equation. Phys. Rev. B.

[34] Xia F., Cui S., Pu X. (2022). Performance study of foam ceramics prepared by direct foaming method using red mud and K-feldspar washed waste. Ceram. Int..

[35] Long Y., Song Y., Jia J., Tang L., Shen D., Gu F. (2024). Preparation of foam glass ceramics by sintering of hazardous waste vitrification slag and biochar. JOM.

[36] Ren P., Zhou R., Liu H., Huo Y., Wang Y. (2024). Preparation of coal gangue based foamed ceramics with SiC as blowing agent and study on its thermal insulation performance. Ceram. Int..

[37] Dong Y., Jiang C., Zhang L., Wang D., Huang S., Cheng X. (2022). Waste-bearing foamed ceramic from granite scrap and red mud. Int. J. Appl. Ceram. Technol..

[38] Chen S., Luo L., Sun H., Peng T., Lei J., Wu M., Wang C., Huang S. (2023). Effect and mechanism of Fe_2_O_3_ decomposition in the preparation of foaming ceramics from industrial solid waste. Int. J. Appl. Ceram. Technol..

[39] Fu F., Hu N., Ye Y., Chen G., Jia J. (2023). Production of lightweight foam ceramics by adjusting sintering time and heating rate. Constr. Build. Mater..

[40] Hu S., Li D., Li Y., Guo Q., Tian D., Zhang L., Li H. (2023). Preparation of foamed ceramics from graphite tailings using a self-foaming method. Minerals.

